# Understanding Surface Adhesion in Nature: A Peeling Model

**DOI:** 10.1002/advs.201500327

**Published:** 2016-01-25

**Authors:** Zhen Gu, Siheng Li, Feilong Zhang, Shutao Wang

**Affiliations:** ^1^ Laboratory of Bio‐Inspired Smart Interface ScienceTechnical Institute of Physics and ChemistryChinese Academy of SciencesBeijing100190PR China; ^2^ Beijing National Laboratory for Molecular Sciences (BNLMS)Key Laboratory of Organic SolidsInstitute of ChemistryChinese Academy of SciencesBeijing100190P. R. China; ^3^ University of Chinese Academy of SciencesBeijing100049P. R. China

**Keywords:** surface adhesion, peeling model, surface topography, interfacial linker

## Abstract

Nature often exhibits various interesting and unique adhesive surfaces. The attempt to understand the natural adhesion phenomena can continuously guide the design of artificial adhesive surfaces by proposing simplified models of surface adhesion. Among those models, a peeling model can often effectively reflect the adhesive property between two surfaces during their attachment and detachment processes. In the context, this review summarizes the recent advances about the peeling model in understanding unique adhesive properties on natural and artificial surfaces. It mainly includes four parts: a brief introduction to natural surface adhesion, the theoretical basis and progress of the peeling model, application of the peeling model, and finally, conclusions. It is believed that this review is helpful to various fields, such as surface engineering, biomedicine, microelectronics, and so on.

## 1 Introduction

**Figure 1:**
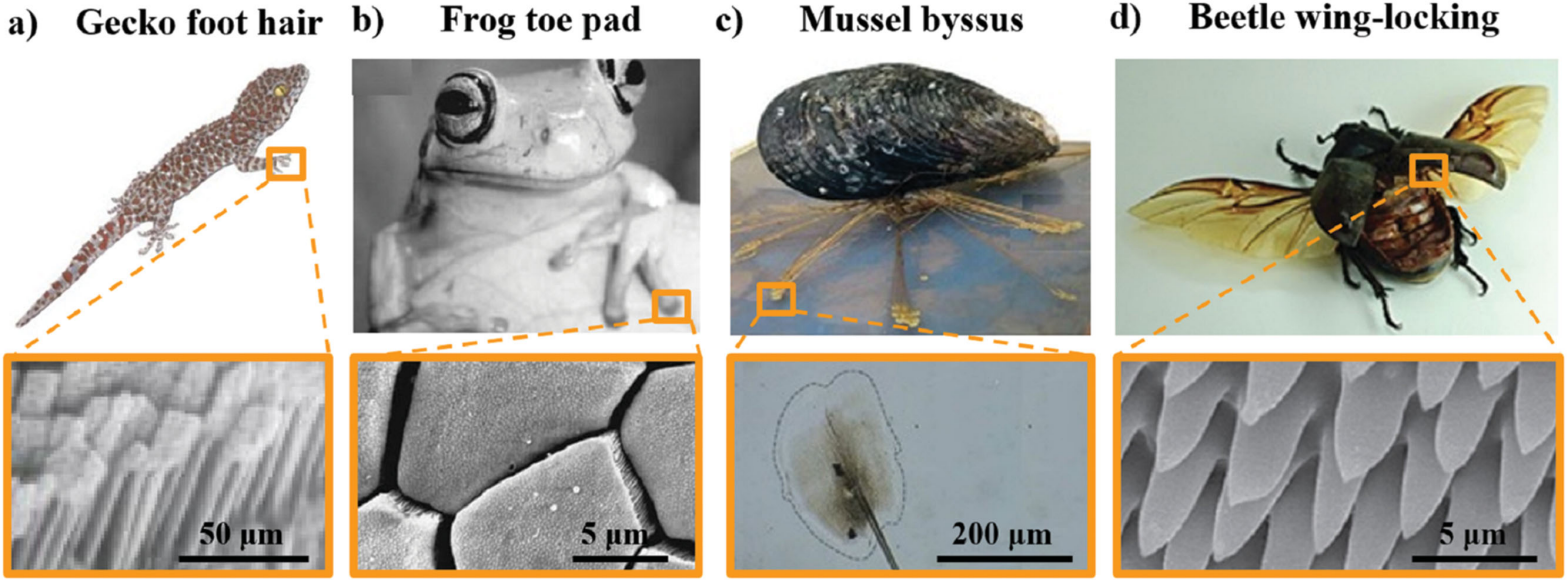
Biological adhesive examples including the creatures and their adhesive structures. a) Gecko toe pads demonstrate reversible adhesive, superhydrophobic, and self‐cleaning properties owing to the aligned microsetae splitting into hundreds of nanospatulae. Adapted with permission.^2^ Copyright 2000, Nature Publishing Group. b) Tree frog toe pads exhibit reversible adhesion under wet conditions originating from soft patterned pads separated by channels at nano/micro scales and mucus secreted from glands. Adapted with permission.^7^ Copyright 2006, The Royal Society. c) Mussel byssus firmly stick to nearly all surfaces underwater due to a set of threads, which were each composed of a terminal adhesive plaque. Adapted with permission.^12^ d) Beetle wing‐to‐body interlockers show reversible binding arising from slightly bent microhairs in one direction with highly reversible adhesion. Adapted with permission.^15^

After 4.5 billion years of evolution and intrinsic selection, many natural surfaces in biological system exhibit numerous amazing and unique adhesive properties.^1^ These unique properties arise from ingenious surface topography and chemical composition. For instance, gecko can easily and reversibly stick to various surfaces due to the fibrillar micro/nano structures (microsetae and nanospatulae) (**Figure** **1**a), while structural integrity can be maintained well after detachment.^2^, ^3^, ^4^ Tree frog (Figure 1b) can climb on tree in wet environments without falling. It is revealed that the hydrophilicity, micro‐scaled channels and protrusions, and the secretion of toe‐pad surface mainly contribute to the wet adhesion.^5^, ^6^, ^7^, ^8^, ^9^, ^10^, ^11^ In the sea, mussels are capable of attaching to any solid surfaces (Figure 1c). The structure and composition of their byssuses are exquisitely adapted for the underwater adhesive properties. Architecturally, the byssuses are hierarchically organized with a leathery bundle of threads tipped by flattened adhesive plaques. Those plaques are composed of at least eight different types of mussel foot proteins (mfps). The catecholic amino acid 3,4‐dihydroxyphenylalanine (Dopa) is the important ingredient of mfps. The catechol moieties of Dopa robustly adhere to the rocks by chelation with the inorganic oxides.^12^, ^13^, ^14^ Interestingly, beetle's reversible wing‐to‐body adhesion originates from slightly bent microhairs interlockers. The dense microhair arrays are distributed on anterior field of thorax (Figure 1d) and utilized to attach the wing by a high shear locking force but an effortless normal lift‐off. This wing‐to‐body interlocker is also able to withstand numerous cycles of reciprocating motion while need no extra applied load or specific surface.^15^

These amazing phenomena in natural surface adhesion have attracted much attention to explore their underlying adhesion mechanisms. Among various adhesion models^16^, ^17^, ^18^ attempting to describe the actual adhesion, the peeling model has been developed to understand surface adhesion of ants,^19^ bees,^19^ flies,^20^ cockroaches,^21^ geckos,^2^, ^22^ frogs,^8^ spiders,^23^ and so on. As peeling model is approaching to describing the attachment and detachment processes of two adhesive surfaces accurately, it is being regarded as the theoretical foundation to design surface adhesive materials including modulating the topography of two surfaces,^24^, ^25^, ^26^ adding the interfacial linkers.^27^, ^28^, ^29^ What is more, peeling is an interesting and ubiquitous process in biological and artificial adhesive systems. For example, peeling is important in the attachment and detachment processes of artificial tapes used to fix objects and biological adhesive systems including ants, bees, flies, cockroaches, geckos, frogs, and so on.^2^, ^8^, ^19^, ^20^, ^21^, ^22^, ^48^ Peeling is also an important phenomenon in many applications, such as paint and coating technology, cell adhesion, and transfer printing.^23^, ^30^, ^48^ Therefore, it is essential and urgent to overview the recent advances in peeling model for surface adhesive systems. After introducing the theoretical background and progress of peeling model, we discuss how the peeling model effectively aids in understanding biological and biomimetic surface adhesion and designing surface adhesive materials. Finally we make a brief summary and an outlook on the peeling model in future research.

## 2 Theoretical Background and Progress of the Peeling Model

The peeling model is presented to understand the process of peeling off one surface from another under a certain peel angle. Based on the nature of the interacting surfaces, the forces connecting two surfaces are mainly classified into four types: surface and field forces, material bridges, mechanical interlocking, and suction forces.^31^, ^32^ Depending on different surface topography, four peel test methods could be employed to evaluate adhesion forces: variable angles and 90° peel tests, wedge peel test, twist test, and T‐peel test. In order to describe the peeling behavior of surface adhesion, peeling model is developed which will be discussed in detail in this Section.

### 2.1 Different Types of Adhesive Force

**Figure 2:**
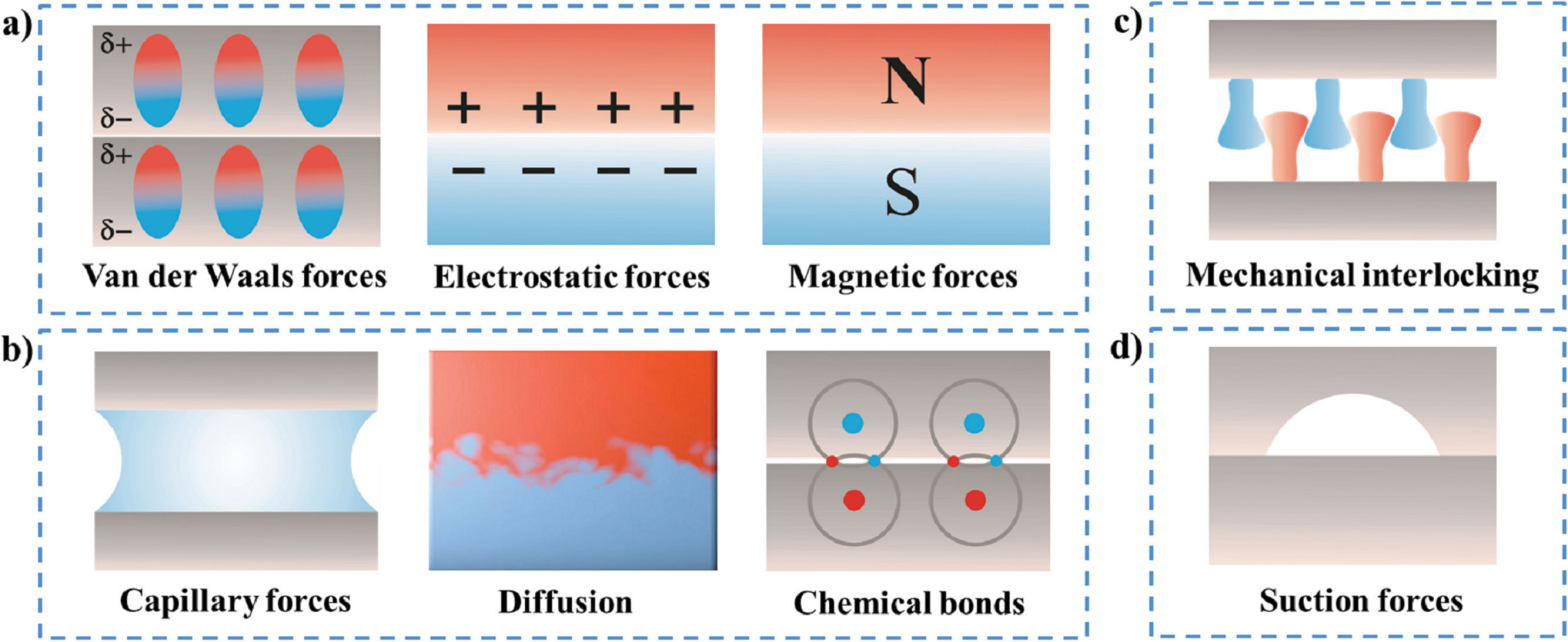
Description of different types and characteristics of main adhesive interactions: a) surface and field forces, b) material bridges, c) interlocking by shape effects, d) suction forces. Adapted with permission.^52^ Copyright 2014, Annual Reviews.

Generally, adhesion involves two interacting surfaces and the type of dominant interaction between two surfaces is determinative for the adhesive behavior. Depending on the essence of the interacting surfaces, the adhesive forces are classified into four main types: surface and field forces (such as van der Waals forces, electrostatic forces, and magnetic forces), material bridges (such as capillary forces, chemical bonds, and diffusion), mechanical interlocking, and suction forces.^31^, ^32^ The main adhesive forces are schematically described in **Figure** **2**.

The van der Waals forces (Figure 2a) generally include Keesom force (dipole‐dipole interaction), Debye force (dipole‐induced dipole interaction), and London dispersion force (induced dipole‐induced dipole interaction). Since the London interaction is always present, the van der Waals forces exist for all contact systems.^33^ The forces are not easily felt due to their short range about 0.2–40 nm.^34^ However, as a famous example in nature, the gecko toe pads utilize the van der Waals forces to get strong and reversible adhesion to substrate.^2^

Electrostatic forces (Figure 2a) are similar to the van der Waals forces in essence.^35^ They are the attractive or repulsive forces between two surfaces with opposite or same charges.^36^ However, compared to the van der Waals forces, electrostatic interactions may be dominant even at larger distance about several micrometers.^37^, ^38^ The electrostatic forces are usually much higher than the van der Waals forces. For example, the electrostatic forces are 8–17 times larger than the van der Waals forces for the silica and mica thin film contact (the radius is 1.5 cm).^39^ Besides, the van der Waals forces were considered as the main interactions resulting in the bio‐inspired dry adhesion. However, recently, a few experiments found electrostatic forces were thought to mainly dedicate the strength of the dry adhesion.^40^, ^41^

Similar to electrostatic forces, magnetic forces (Figure 2a) are inversely proportional to the cube of the separating distance between magnetic dipoles.^42^ Magnetic forces are much weaker than electrostatic forces between molecules. However, the larger molecules or particles become, the more important are magnetic forces,^34^ because magnetic interaction energy increases with the volume of molecules or particles and is cube proportional to the diameter of molecules or particles. While electrostatic interaction energy is inversely proportional to the diameter.^34^ Magnetic forces are widely used to drive self‐assembly of particles,^43^ actuate artificial cilia,^44^ and so on.

Capillary forces come from the liquid in the gap between two surfaces, which contribute to the total adhesion greatly (Figure 2b).^45^ In general, capillary forces can be explained by the theory of wetting and thermodynamic adsorption. A good wettability means that the liquid and solid have a strong affinity and adhere well.^46^ Capillary forces exist in most cases of “stiction” between the small constituents in microelectronics equipments.^47^

Diffusion (Figure 2b) is mainly applicable between two miscible polymers by entanglements of chains.^48^ It is widely used in the researches of self‐healing polymer,^49^ toughness of the interface,^50^ and so on. It is difficult that atoms or molecules of metal and ceramic systems diffuse across an interface. Thus, diffusion needs heating such as sintering of metal or ceramic powders.^51^

Chemical bonds (Figure 2b) result from electrostatic attraction and repulsion among electrons and nuclei. They include ionic bonds, covalent bonds, and metallic bonds. Chemical bonds are short‐range interactions, for example, the physical length of covalent bonds is 0.1–0.2 nm.^34^ Although chemical bonds are usually very strong (about 100–1000 kJ mol^−1^),^48^ they need two surfaces with special chemical and physical properties.^52^

Mechanical interlocking (Figure 2c) is another force resisting the separation between two solids. Such interlockers can be widely applied without considering surface properties and environmental conditions. Some biological surface structures serve the fixation of the surfaces,^53^, ^54^ for example, the hook‐like surface structures of some plant fruits, the head‐to‐body interlockers of dragonflies, and the wing‐to‐body interlockers of beetles. In practical applications, fastening systems and three‐dimensional structures are utilized. The simplest example is fixation of two solids which are interlocked with each other by a screw.^55^

Suction forces (Figure 2d) are calculated through multiplying the projected area of the suction cup by the pressure difference between the internal and surrounding pressures.^56^ Some creatures utilize suction forces to adhere to substrates such as octopus suckers^57^, ^58^ and climbing fish oral suckers.^59^ There is dispute within suction forces. For example, the underwater adhesive strengthes of mushroom‐shaped and hexagonal pillar microstructures are much higher than that of flat surfaces.^60^, ^61^ Suction forces^60^ and capillary/direct contact forces^61^ are thought to play the key role respectively.

### 2.2 Different Contact Geometric Arrangements

**Figure 3:**
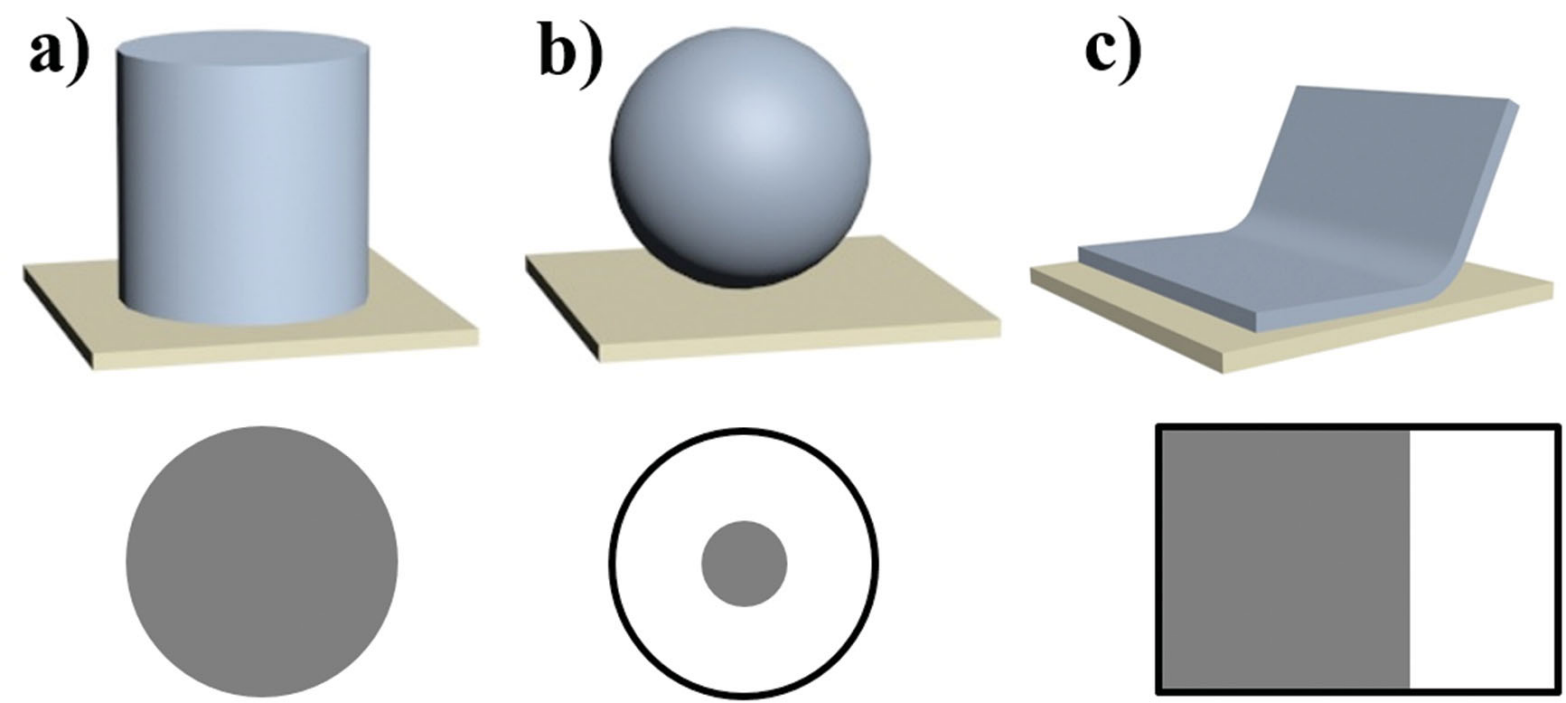
Schematic of different contact geometric arrangements: a) “flat‐on‐flat” configuration, b) “ball‐on‐flat” configuration, c) peeling configuration, (in each case, the shadow denotes contact area). Adapted with permission.^32^

Natural attachment systems exhibit various contact shapes.^2^, ^7^, ^15^ When treating these geometries theoretically, Spolenak et al. found that different contact geometric arrangements greatly influenced the characterization of adhesion.^32^, ^62^ The geometric arrangements can be mainly classified into three types (**Figure** **3**): the “flat‐on‐flat” configuration (Figure 3a), the “ball‐on‐flat” configuration (Figure 3b), and the peeling configuration (Figure 3c).^63^, ^64^, ^65^

The “flat‐on‐flat” configuration (Figure 3a) is the best arrangement and presents the highest adhesive strength between two ideally smooth surfaces. However, it is sensitive to even small roughness.^62^, ^66^ The widespread use of “ball‐on‐flat” (Figure 3b)^17^, ^67^, ^68^, ^69^, ^70^, ^71^ overcomes misalignment problem inherent to flat‐on‐flat geometry. Just like setae found in some insects, spiders, and geckos,^72^, ^73^ researchers roughly split one solid of the smooth, continuous adhesive contact systems such as the “flat‐on‐flat” and the “ball‐on‐flat” geometries into subsets that have evolved numerous hairlike adhesive setae. This fibrillar type consists of many smaller subcontacts. When preloads attach fibrillar arrays to the substrate, each fiber begins to resemble peeling geometry which is peeling off one surface from another under a certain peel angle (Figure 3c). We will discuss this model in more detail in the following part.

### 2.3 Different Peel Test Methods

**Figure 4:**
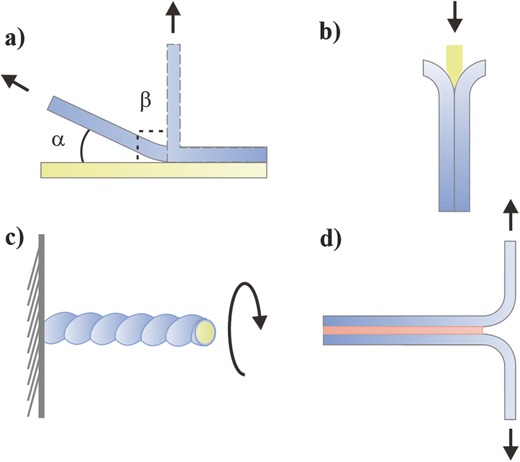
Four main different peel measurements: a) variable angles (*α*) and 90° (*β*) peel test, b) wedge peel test, c) twist test, and d) T‐peel test.

To meet different situations of surface contact,^74^, ^75^, ^76^, ^77^, ^78^, ^79^, ^80^ four kinds of methods based on peeling model have been developed to characterize the surface adhesive force (**Figure** **4**). The most commonly used method is that peeling one end of the sample from the substrate at a certain angle (Figure 4a). During peeling, stress is applied at a line; test loads are expressed in force density, namely, stress per unit width. Among peeling at variable angles, the 90° peel test is the most common test for detecting the peel strength. For example, Majumder et al.^81^ embed fluid‐filled microchannels within flexible plates bonded to a substrate. The 90° peel strength is enhanced by subsurface microstructures due to the crack‐arresting and surface stresses. Ghatak et al.^77^ peel a film with periodically varying modulus along the peeling direction under 90°. The peel strength is enhanced due to the intermittent progression of the peeling front. Furthermore, peeling at different angles (α) (Figure 4a) are also important for peeling measurements. For some animals such as bees,^19^ flies,^20^ cockroaches,^21^ geckos,^2^, ^22^ frogs,^8^ and spiders,^23^ the angle between the detachment force vector and the surface has a significant influence on pad adhering and/or detaching. On the other hand, the peeling process at angles greater than a critical angle may not involve any breaking of the tape on the substrate, and the tape can be reused many times without damage.^75^

The second method is the wedge peel test (Figure 4b) to evaluate the adhesive strength in mechanical manufacturing field. During the test, a wedge, in touch with two adhesive surfaces, is drawn along the length direction of the specimen.^78^, ^82^ The values of peel strength measured using wedge peel tests, corresponded with the results by fracture mechanics methods.^78^

The third method is the twist peel test (Figure 4c) to qualitatively characterize the adhesive strength of coating in the winding wire industry.^79^ It is performed by fixing one end of the wire to an anchor, then rotating the other end along the diameter direction at a certain speed until the coating is detached. The rotation number represents the peel strength. Although the twist peel test can't quantitatively provide the peel strength, it does allow a comparison among different samples.^79^

The fourth method is the T‐peel test (Figure 4d) to assess the adhesive strength of organic coatings and adhesive joints.^83^, ^84^, ^85^, ^86^ In some cases such as polyimide coatings on flexible substrate, the T‐peel test is more suitable for measuring the adhesive strength than the 90° peel test.^84^

### 2.4 Important Theoretical Progress of Peeling Model

**Figure 5:**
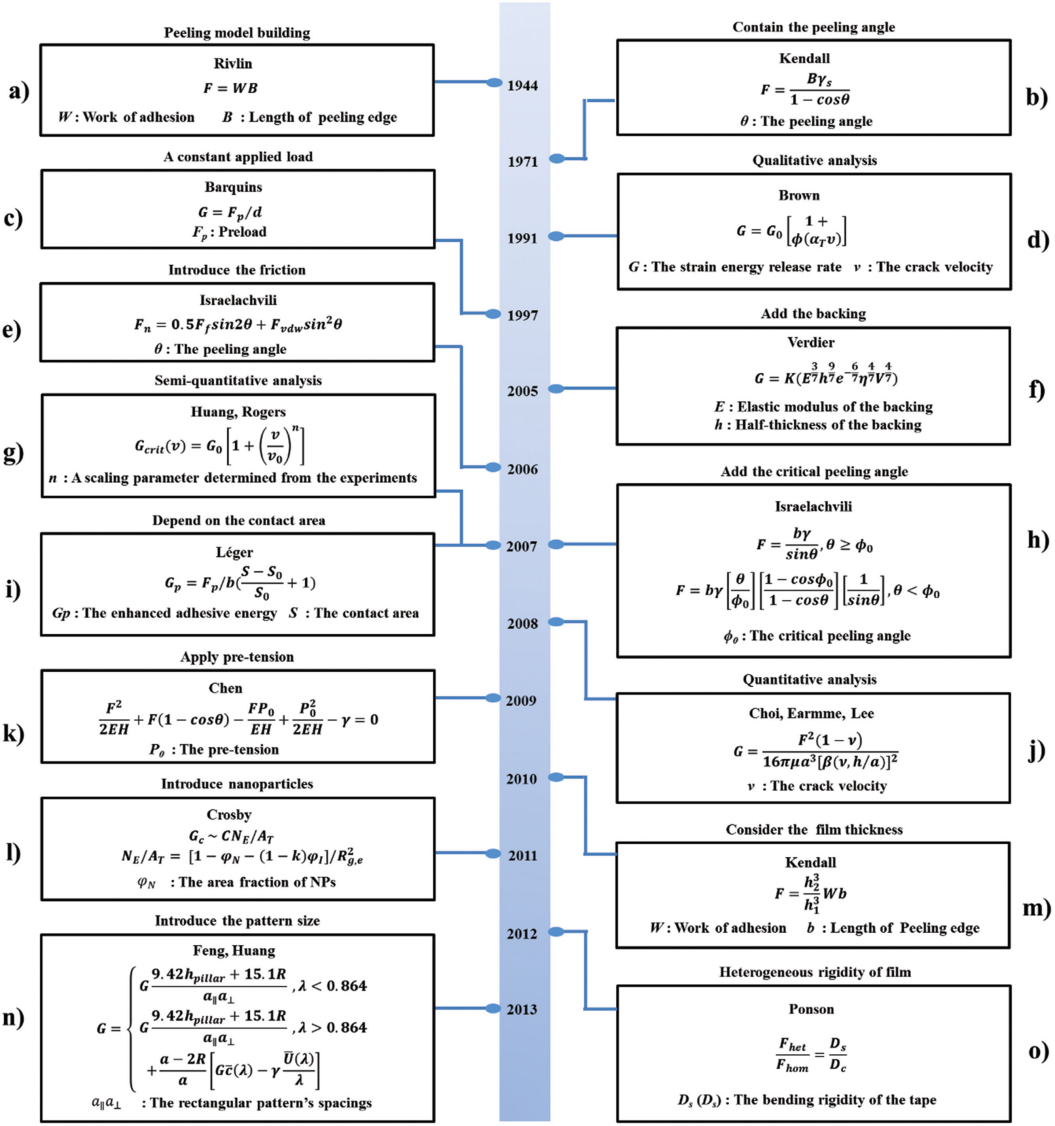
Timeline for peeling model theory research from 1944 up to now. a) Peeling model is first proposed by Rivlin.^87^ b,h) Peel forces depend on peel angles.^88^, ^89^ d,g,j) Peel forces depend on peel velocity.^90^, ^91^ Peel forces depend on c) applied load,^92^ e) friction,^93^ and k) pre‐tension.^94^ i,n,o) Peel forces depend on heterogeneous topographical and mechanical properties.^95^, ^96^, ^97^ f,m,l) Peel forces depend on backing and linker.^28^, ^98^, ^99^

Various models have been developed to explain the peeling properties between two surfaces.^87^, ^100^, ^101^, ^102^, ^103^, ^104^, ^105^, ^106^, ^107^ The most popular model was first proposed by Rivlin^87^ (**Figure** **5**a). As the simplest peeling model, however, Rivlin's equation only focuses on the transverse size of the film and the adhesion energy. Considering the viscous processes of the surface adhesion,^88^, ^108^ complex loading conditions,^109^ and diverse adhesive structures in dry^110^, ^111^, ^112^ and wet,^112^, ^113^ researchers gradually introduced a series of parameters into the model proposed by Rivlin, such as velocity,^73^, ^74^, ^78^, ^79^, ^87^ preload,^50^, ^92^ properties of the backing and substrate,^89^, ^98^ and so on, to make peeling model more effectively reflect the adhesive property between two surfaces.

Firstly, Kendall (1971)^88^ (Figure 5b) introduced the peel angle (*θ*) and the surface energy of the solid (*γ*_S_) into modeling peel force. Considering the topography of the peel‐zone, Pesika et al.^89^ further added a critical peel angle (*φ*_0_) to the Kendall equation (called the peel‐zone model, short for PZ model) (Figure 5h). The critical peel angle (*φ*_0_) is used to distinguish two peel regimes. When *θ* is larger than *φ*_0_, it is the constant peel‐zone regime, and the peel force is invariable with different peel angle. When *θ* is smaller than *φ*_0_, it is the variable peel‐zone regime, and the peel force is increased with decreasing peel angle.

However, peel forces usually vary with different peeling velocities. Brown et al.^50^, ^114^ (Figure 5d) qualitatively explained the velocity (*v*) dependence of the strain energy release rate (*G*) that was related to peel force. The function between *v* and *G* can't be given as a quantitative expression. The relation is influenced by the Williams Landel Ferry shift factor, which can be obtained by shifting various temperatures to a reference temperature. Then, for both high and low peeling velocities,^115^, ^116^, ^117^, ^118^ a large temperature scales^115^, ^117^ and either metal/polymer or polymer/polymer contacts,^90^, ^92^, ^117^, ^118^, ^119^ researchers have revealed the semi‐quantitative power law relation between the peeling velocity and peel force (Figure 5g). The exponential value comes from experimental data. The scaling parameter values are variable for different adhesive materials. In addition, Choi et al.^91^ quantitatively analyzed the dependence of adhesive strength on the detachment velocity (Figure 5j) by extending Kendall's theory of adhesion.

Peel force also depends on the applied load (Figure 5c),^92^ friction (Figure 5e),^93^ and pre‐tension (Figure 5k).^94^ On one hand, the preload dependence of the peel force is attributed to viscous processes of surface adhesion.^88^, ^108^ On another hand, Tian et al.^93^ theoretically analyzed the function of friction on the peel force in a peeling model (Figure 5e) and explained the firm attachment and easy detachment of gecko foot. They considered the pulling force had two parts: a ‘‘normal adhesive force'' from the interaction zone between two surfaces, and a ‘‘lateral friction force'' at the segment of the film still in touch with the surface. To attach, the net frictional and adhesive forces on the entire film are rapidly increased to a very high value by rolling the film downward and forward. To detach, the very low frictional and adhesive forces are quickly obtained through rolling the film up and back.^93^ Chen et al.^94^ found that at small peel angles, the pre‐tension could greatly enhance the peel strength; whereas at large peel angles, the pre‐tension could decrease the peel force. So, they can get a strongly reversible adhesion (Figure 5k). The critical angle is increased with the parameter *γ*/*EH*, where *γ* is the van der Waals interaction energy between two surfaces, *E* is the Young's modulus of the film, *H* is the thickness. The expression has been verified by the previous experiment. Schubert et al.^120^ found the value of the critical angle was decreased with the increasing modulus: the polymer microfibre arrays with higher modulus correspond to the lower value of critical angles, while the lower modulus exists higher critical angles. What is more, when the pre‐tension beyond a critical value, $P_{0}\;>\;\sqrt{2\gamma EH}$, where *P*_0_ is the pre‐tension, the peel force drops sharply at the critical peel angle.^94^ The experimental result consists with the discovery of the gecko's detachment. A live gecko mostly easily peels toes off from the substrate at a critical detachment angle.^121^

The above mentioned models are focused on homogeneous surfaces which can't explain the peeling properties on heterogeneous surfaces. This part summarizes that the topography of heterogeneous surface, elastic heterogeneity, and the pattern size all influence the peel force. For topographic heterogeneity, according to the research of Lamblet et al.,^95^ there is no obvious difference in the peel energy between patterned and smooth surfaces, while the peel force on the substrate with patterned surface is higher than that on the smooth surface. The peel force on the patterned elastomer can be calculated on the basis of the force detected from a smooth substrate (Figure 5i). The reason for the enhancement is that the patterning enlarges the real touch area between the two surfaces. For example, the elastomer may invade the gaps in the pattern. Then, Chen et al.^97^ found that with increasing pattern spacing, the peel force was decreased and became even lower than that at a flat surface (Figure 5n). However, when the pattern spacing reached a threshold distance, the peel force jumped suddenly to significantly higher value (Figure 5n) due to the patterned surfaces collapsing to the substrate which results in a larger contact area. Huang et al.^122^ have investigated self‐collapse. The cause of the collapse is that the strip/substrate adhesion energy is higher than the deformation energy of the strip collapse above the critical pattern spacing value. Besides, Chen et al.^97^ analytically calculated the energy release rate of the pattern strip (Figure 5n). They also found the analytic results were consistent with the experimental data. However, the function (Figure 5n) is only applicable to patterned cylindrical pillars. For elastic heterogeneity, Xia et al.^96^ showed that the peel force was enhanced by patterning the elastic bending stiffness of the film due to the fluctuations of the bending energy. That is to say, for patterned elastic properties, when the peeling front crosses from a stiff to a compliant region, the bending energy is increased rapidly; on the other hand, as the front traverses from the flexibility to the stiffness, the bending energy falls quickly. The rapid variation of the bending energy increases the whole adhesion energy and enhances the peel force. Further, the enhancement ratio depended on the ratio of the bending rigidity between the stiff and compliant region (Figure 5o). Xia et al.^96^ showed the overall adhesion energy was regulated by the sites with large adhesion energy. The arrangement of the pinning sites can be used to design anisotropic and asymmetric adhesive systems.

The fraction of nanoparticles and the properties of the backing,^28^, ^98^, ^99^ such as the elastic modulus and half‐thickness, were also introduced into the peeling model (Figure 5m,l). For example, Su et al.^28^ found that the peel strength was first enhanced and then decreased with increasing tailored nanoparticle content (Figure 5l). This phenomenon can be explained from the glassy polymer‐nanoparticle composites. The optimal fraction of nanoparticles increases the peel strength based on the balance of two mechanisms: nanoparticles restricted in the craze decrease 1) the amount of cross‐tie fibrils and 2) the extensibility of the craze.^123^ Kendall et al.^99^ found the third power law relation of the peel force with the film thickness (Figure 5m).

### 2.5 Advances in Simulations of the Peeling Model

**Figure 6:**
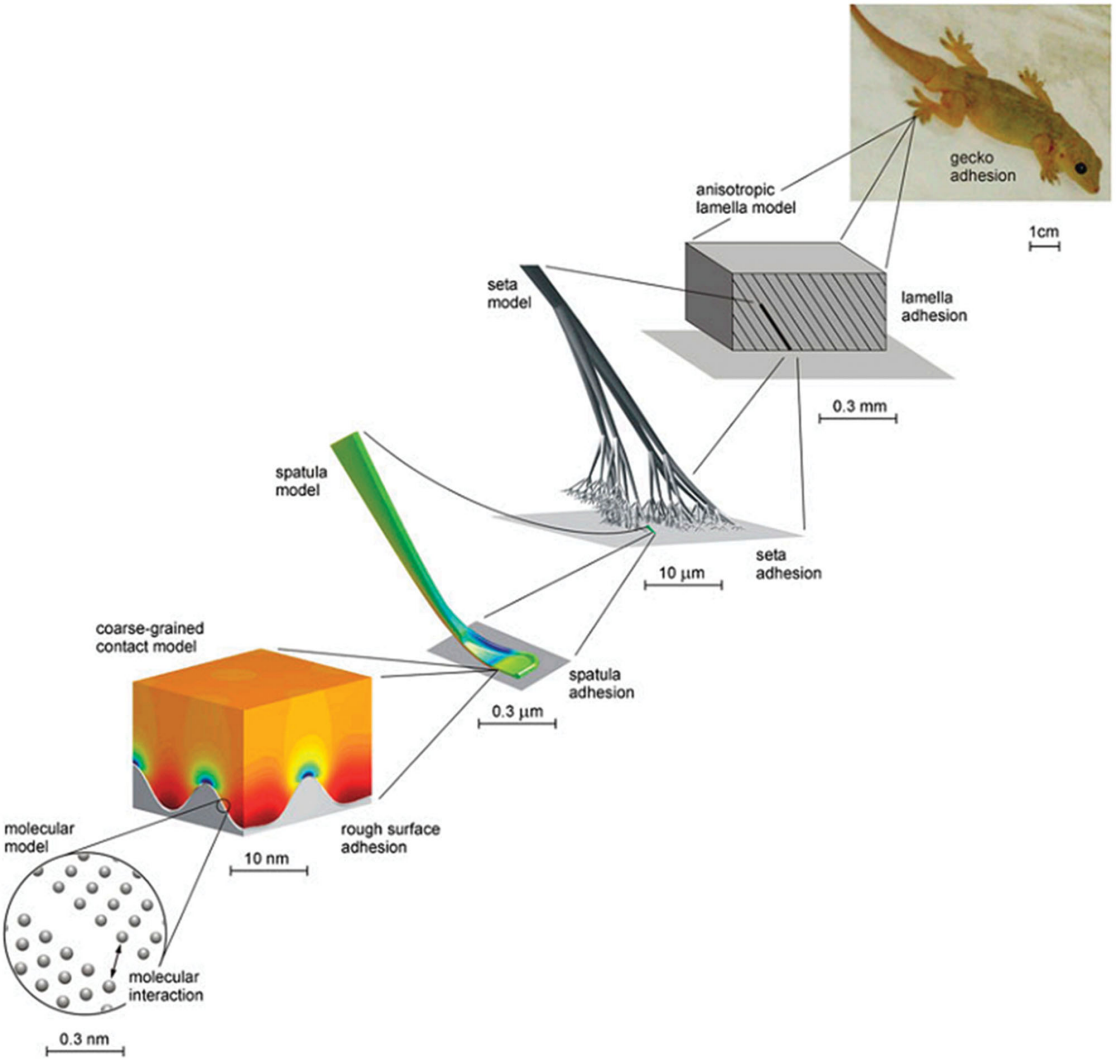
Multiscale simulation of the gecko hierarchy adhesion behavior. Reproduced with permission.^131^ Copyright 2012, Taylor & Francis.

Although the above various analytical models have been developed to make peeling model more effectively reflect the adhesive property between two surfaces,^87^, ^100^, ^101^, ^102^, ^103^, ^104^, ^105^, ^106^, ^107^ analytical models become extremely complex for hierarchical architectures^124^ and neglect the detailed peeling process.^125^ In such cases, numerical methods are appropriate.^124^, ^126^, ^127^ For example, using molecular dynamics simulation, Chen et al.^128^ investigated the process of peeling off a graphene sheet from a corrugated surface. The roughness of corrugated surface exerts great influence on the peeling process. With finite element simulations which serve as the most common method,^126^ Zhao et al.^129^ demonstrated a hierarchical wavy interface could improve the peel strength. Sauer^130^ multiscalely simulated the adhesion of a gecko seta at three length scales, including the branched structure of the gecko seta with a few micrometers (a finite element simulation), the spatulae adhesion with a few nanometers (another finite element simulation), and the rough surface adhesion with several angstroms (molecular interaction potential). Recently, Sauer^131^ reviews the advances in the simulation of the gecko adhesion behavior at different length scales (**Figure** **6**), ranging from a few angstroms to several centimeters.

## 3 Application of the Peeling Model

As discussed above, the peeling model is gradually developed to approach the real adhesion events. Natural^2^, ^8^, ^19^, ^20^, ^21^, ^22^ and biomimetic^132^, ^133^, ^134^ surface adhesion can be easily understood with peeling model. For instance, the directional adhesion of some creatures can be explained by considering the peel angles of the animals pads to the substrate, and peeling model also gets further development. Then, researchers can design the artificial surface adhesion by changing the backing and substrate topographies or adding interfacial linkers. Topographies of two surfaces and interfacial linkers may change the contact line and/or adhesion energy which are closely related to the surface adhesion behaviors.

### 3.1 Peeling Model for Understanding Natural and Biomimetic Surface Adhesion

In the peeling model, the directional adhesive properties can be realized by tailoring the angle between the peel force vector and the substrate, even with no need for any apparent orientation of the adhesive structure itself. Corresponding to a low peel angle, the peel strength is high, while the easy detachment is realized at a high peel angle.^89^ Researchers could apply this idea to study natural^2^, ^8^, ^19^, ^20^, ^21^, ^22^ and biomimetic^132^, ^133^, ^134^ surface adhesion. In the following parts, we will discuss the surface adhesion of gecko, frog, and spider silk in detail.

#### 3.1.1 Surface Adhesion of Gecko Toe Pads

**Figure 7:**
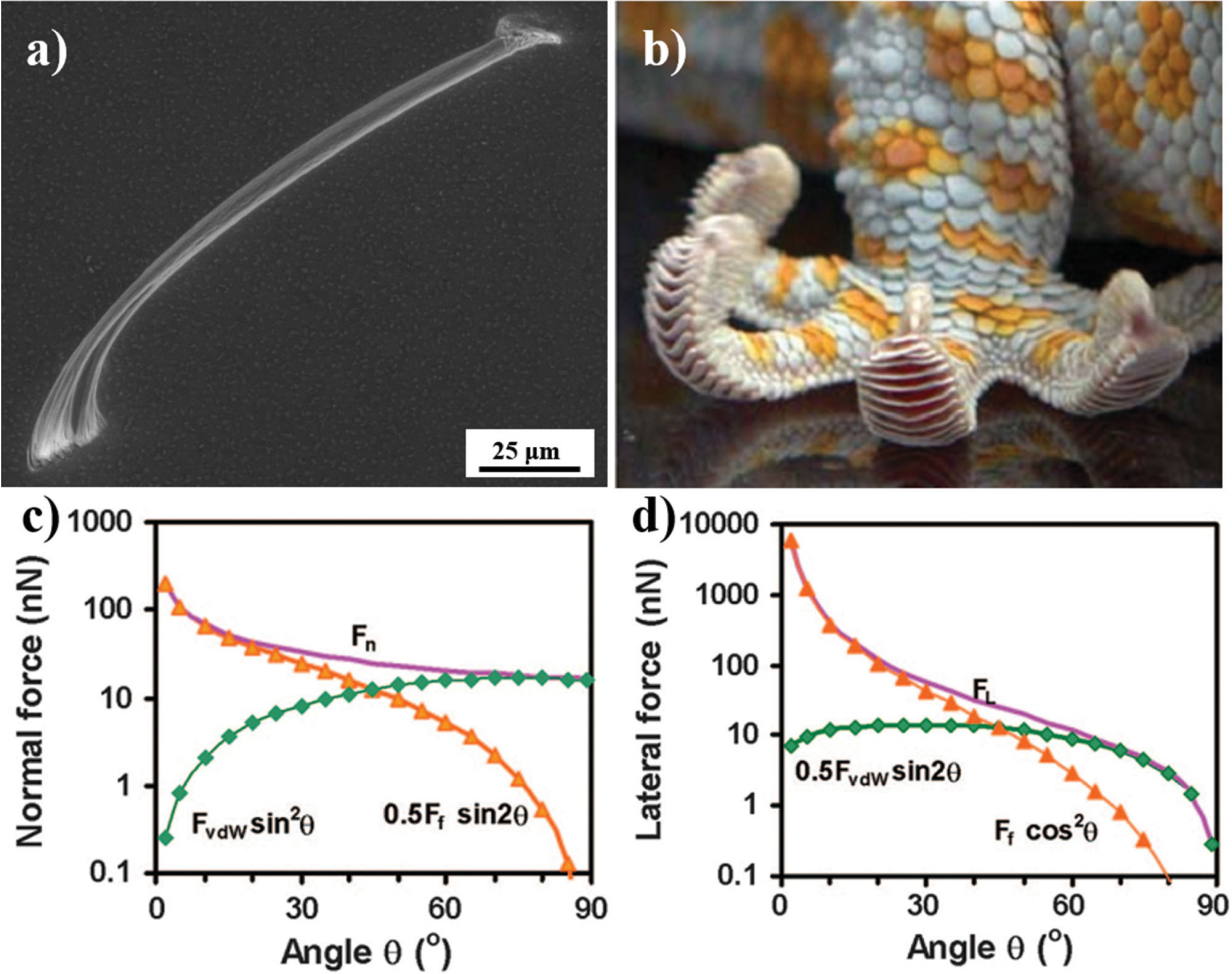
Gecko toe pads surface adhesion. a) A microsized seta is with terminal nanosized spatulae. The adhesion of seta to substratum is regulated by orientation and preloading according to the peeling model. Reproduced with permission.^2^ Copyright 2000, Macmillan Publishers Ltd. b) Low adhesive force are observed by rolling the toes upward and backward to get a large peel angle between the gecko toe and the surface. Reproduced with permission.^138^ c,d) Influence of the pulling angle (*θ*) on the ‘‘normal adhesive force'' (*F*_n_) and ‘‘lateral frictional force'' (*F*_L_), including the contributions of the van der Waals force (*F*_vdW_) and friction force (*F*_f_) to *F*_n_ and *F*_L_. Reproduced with permission.^93^ Copyright 2006, National Academy of Sciences.

Gecko pads amazingly adhere to almost any kind of surfaces to run rapidly on walls and ceilings.^135^ A gecko foot contains about 500 000 microsized setae splitting into nanosized spatulae with a size of approximately 200–500 nm (**Figure** **7**a).^136^ Autumn et al.^2^ discovered two aspects of function regulated the gecko's unique adhesive properties: the special orientation of the toe and preloading.^137^ In addition, on the basis of the peeling model, Tian et al.^93^ analyzed the directional adhesion by assessing the adhesive and frictional forces between the toes and the substrate. A ‘‘normal adhesive force'' (*F*_n_) and ‘‘lateral frictional force'' (*F*_L_) contributed to the pulling force of a spatula. *F*_n_ and *F*_L_ originate from the van der Waals force (*F*_vdW_) and friction force (*F*_f_). The ‘‘normal adhesive force'' and ‘‘lateral frictional force'' both decrease with increasing pulling angles (*θ*) (Figure 7c,d). For example, at angle 10°*F*_n_ and *F*_L_ of a seta are 35 μN and 200 μN, repectively, while at angle 90°*F*_n_ of a seta is 8 μN. So, through rolling down and gripping the toes inward, geckos obtained high net frictional and adhesive forces to adhere to substrate. Whereas geckos peel the spatulas off easily due to the low adhesion/friction by rolling the toes upward and backward (Figure 7b).^93^, ^138^

#### 3.1.2 Surface Adhesion of Frog Toe Pads

**Figure 8:**
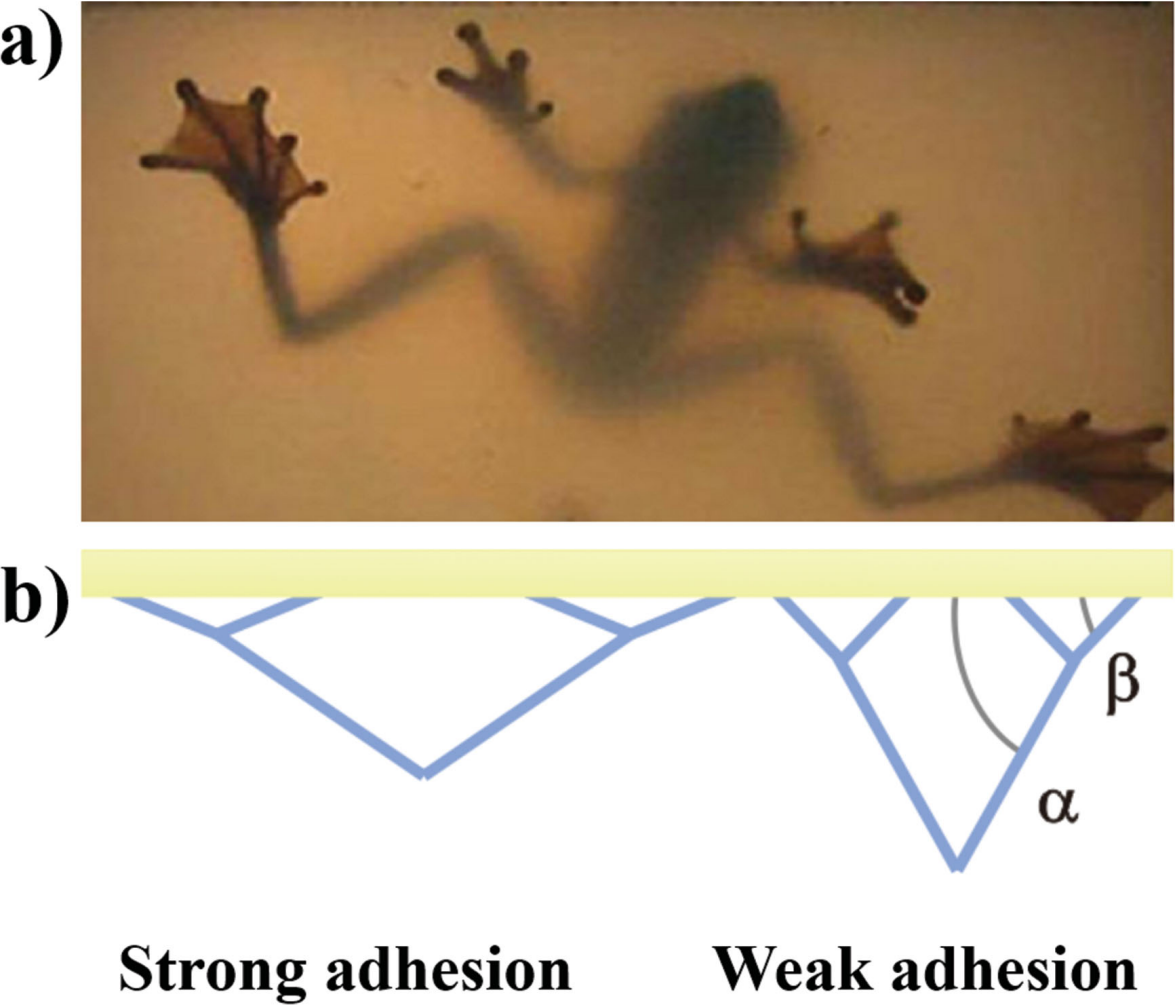
a) Digital photograph of frog toe pads surface adhesion. b) Small leg/substrate and toe/substrate angles correspond to high adhesion force, while large angles lead to low peel force. *α*, leg/substrate angle; *β*, toe/substrate angle. Adapted with permission.^140^ Copyright 2008, European Academy of Sciences.

It is known that several families of frogs adhere in wet condition using expanded adhesive toe pads (**Figure** **8**a) and detach by peeling.^8^, ^139^ According to the peeling model,^100^ Barnes et al.^140^ measured the adhesive forces from single pads pulled off at different angles. They found that spread limbs had relatively small leg/substrate and toe/substrate angles. Thereby, frogs keep firmly adhered to the surface, avoiding peeling (Figure 8). Whereas they can easily peel off from the surface just by increasing leg/substrate and toe/substrate angles (Figure 8b).

#### 3.1.3 Spider Silk Surface Adhesion

**Figure 9:**
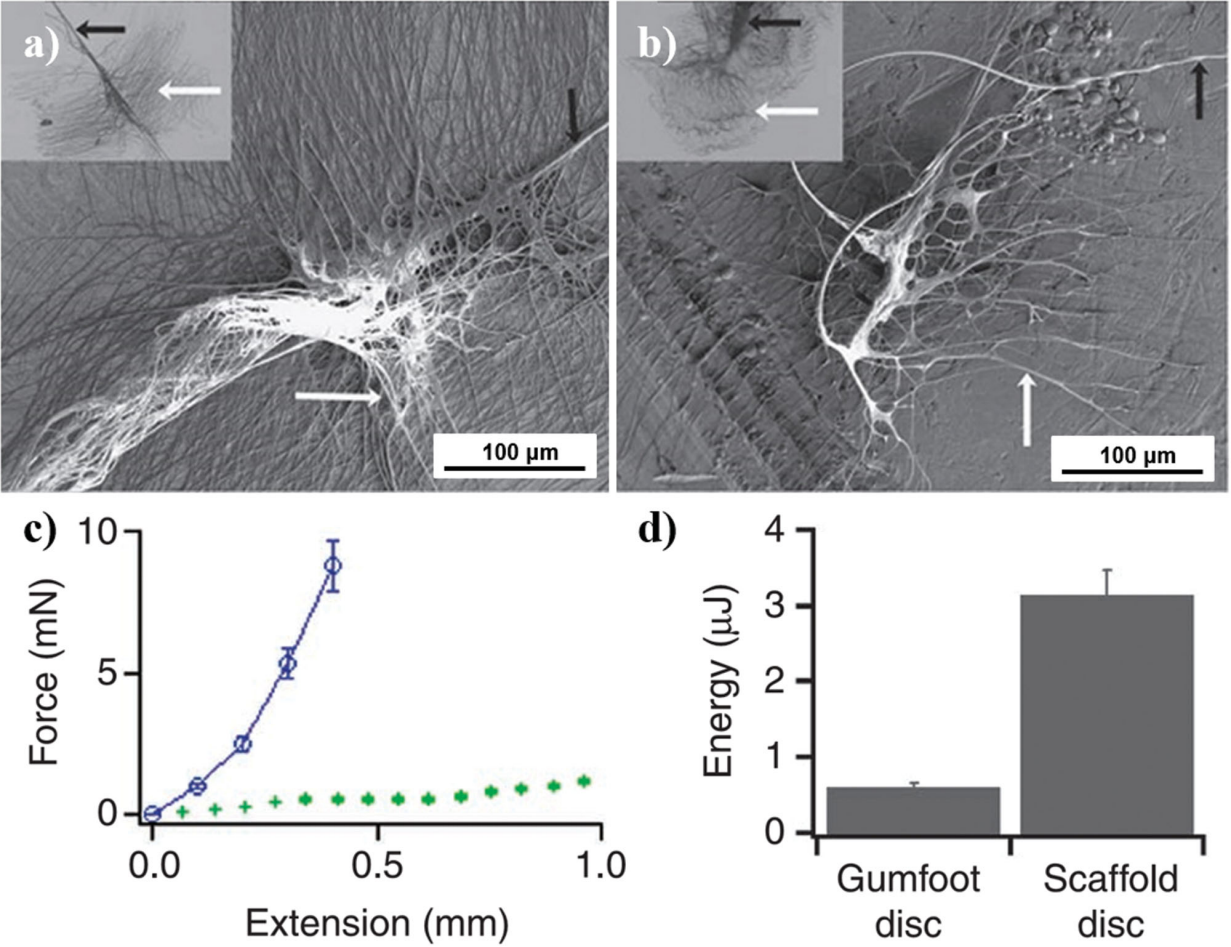
Typical SEM micrographs of a) ‘‘staple‐pin'' architecture and b) ‘‘dendritic'' architecture. Light microscope graphs of the two architectures are shown in insets. In (a) and inset, the black and white arrows point to dragline silk and pyriform fibres, respectively. Pyriform fibres belonging to the scaffolding discs attach the dragline silk to the substrate. In (b) and inset, the black and white arrows denote gumfoot thread and pyriform fibres. Pyriform fibres belonging to the gumfoot discs attach the gumfoot thread to the substrate. c) Force‐extension profiles acquired by peeling off the scaffolding disc (blue) and gumfoot disc (green) from the glass substrate. d) The calculated adhesion energy of the scaffolding disc and gumfoot disc from the force–extension profiles. Reproduced with permission.^141^ Copyright 2012, Macmillan Publishers Ltd.

Spiders ensnare walking and flying prey relying on two types of attachment discs:^100^ scaffolding discs (**Figure** **9**a), strongly attaching the scaffolding silk to the surface, can withstand the impact of prey and entangle flying insects; gumfoot discs (Figure 9b), weakly attaching the gumfoot silk to the substrate, can easily detach from the substrate and capture the insects on the ground. From force‐extension curves (Figure 9c), the calculated adhesion energy of scaffold disc is an order stronger than that of gumfoot disc (Figure 9d). Sahni et al.^100^ explain the divergent adhesive strengths of the two architectures by using Kendall's peeling model. For the scaffolding discs, higher adhesion is due to small peel angles of the pyriform fibres. On the other hand, for the gumfoot discs, relatively lower adhesion is owing to higher peel angles.^141^

#### 3.1.4 Biomimetic Surface Adhesion

**Figure 10:**
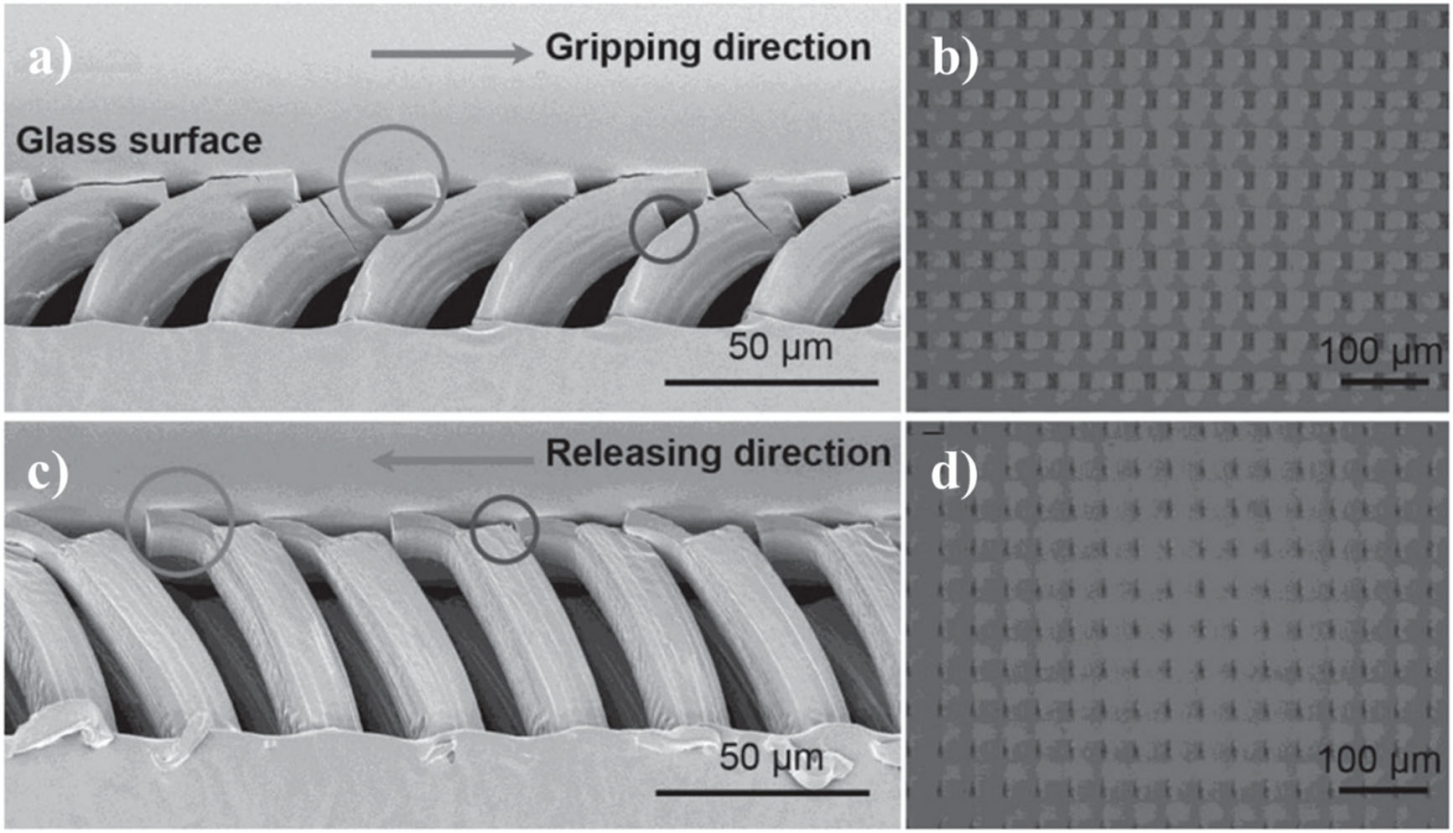
Side‐view SEM micrographs of biomimetic hierarchical structures under shear. a) In the gripping direction, larger circle shows a large contact area and smaller circle indicates the preventation of contact between pillars which is important to prevent clumping of neighboring pillars. c) In the releasing direction, larger circle exhibit a small contact area and smaller circle indicates the preventation of large area contact. b,d) Optical images show the different contacts between the prismatic structures and the transparent glass surfaces in the gripping and releasing direction respectively. Reproduced with permission.^134^

Motivated by the striking switchable adhesion of animals,^2^, ^8^, ^19^, ^20^, ^21^, ^22^ the researchers attempt to design novel adhesive micro/nanostructures.^142^, ^143^, ^144^, ^145^ They often employ the peeling model to effectively understand the unique biomimetic surface adhesion.^132^, ^133^, ^134^ For example, using two‐level gecko‐like prismatic structures (**Figure** **10**), Jin et al.^134^ obtained bidirectional switchable adhesive property. In the gripping direction, the high adhesion is due to the large contact area and low peel angle. In the releasing direction, the low adhesion comes from the small contact area and high peel angle.

### 3.2 Peeling Model for Modulating Artificial Surface Adhesion

Depending on the peeling model, the peel force is proportional to the contact line and two surfaces adhesion energy which can be tailored by changing the backing and substrate topographies or adding interfacial linkers. For example, introducing the triangular wide‐tip micropillars to the backing modulates the contact line by changing the peeling direction angles.^25^ The periodic fibrils topped by a thin plate on the backing enhances the peel strength through periodically variable peel energy.^26^ The patterned surface on the substrate also increases the peel force because of crack nucleation or pinning.^24^, ^146^ Simply introducing interlocking linkers can enhance the peel force resulted from increasing adhesion energy.^27^, ^28^, ^29^

#### 3.2.1 Backing Topography

**Figure 11:**
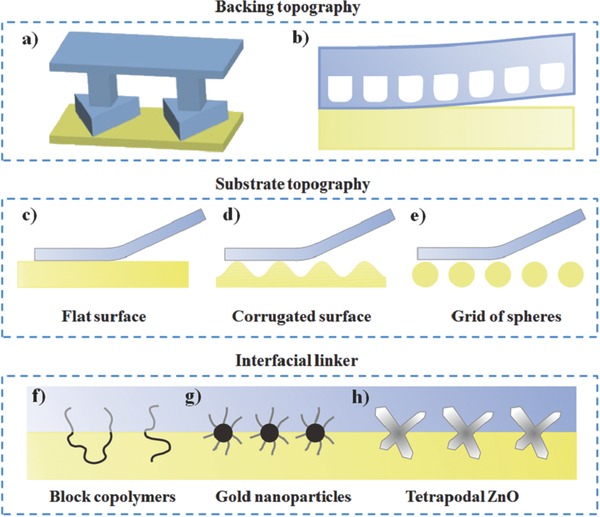
Design of surface adhesion by modulating the backing/substrate topographies and adding the interfacial linkers based on peeling model. a) The unique triangular‐tip geometry exhibits directional dependence of surface adhesion. Adapted with permission.^25^ b) An array of fibrils with a terminal plate enhance the surface adhesion. Adapted with permission.^26^ Copyright 2007, National Academy of Sciences, USA. c,d,e) Various topographies of substrate with flat surface, corrugated surface, and grid of spheres. Adapted with permission.^24^ Copyright 2014, American Physical Society. f,g,h) Design of surface adhesion by adding block copolymers, gold nanoparticles and tetrapodal ZnO. Adapted with permission.^27^ Copyright 2002, American Chemical Society. Adapted with permission.^28^ Copyright 2011, American Chemical Society. Adapted with permission.^29^

On the basis of the peeling model, the peel strength can be tailored by modulating the backing topography to change the propagation of the peeling front^25^ and/or the adhesion energy (**Figure** **11**a,b).^26^ For example, Kwak et al.^25^ described the anisotropic, directional adhesive behaviour of the microsized triangular wide‐tip pillars (Figure 11a). The unique adhesive properties are because that the peeling front propagates differently under varying peel direction angles. The peeling direction angles of 180° and 60° show a large contact line, while a rather short contact is expected for the other angles.^25^ Glassmaker et al.^26^ found that the peel strength could be enhanced by using the periodic fibrils topped by a thin plate (Figure 11b). Because the structure, consisting of the fibrillar array near the interface, will alternately absorb and expel energy. The peel energy will vary periodically as a function of the interfacial crack location within the repeating geometric cell, and then the peel strength is enhanced.^26^, ^147^

#### 3.2.2 Substrate Topography

As discussed in Section **2.4**, the substrate topography regulating surface adhesion strength is significant. Ghatak et al.^111^ found, in comparison with a smooth substrate, the needed force for initiating the crack was higher when the film was peeled off from the substrate with patterned surface. Chung and Chaudhury^148^ considered that the strong enhancement arose from a series of crack nucleation at the heterogenous substrate. From another view, Dalmas et al.^146^ considered the heterogenous substrate induced the crack pinning causing the peel strength enhancement. Chen et al.^149^, ^150^ studied periodic adhesion energy between two nano/micro structured surfaces. There are two parameters controlling the apparent adhesion energy: the period of adhesion energy and the adhesive zone size at substrate. For more systematical analysis, Lindström et al.^24^ studied a patterned surface with nanometer asperities (Figure 11c,d,e). By considering the surface geometry and the film stiffness, they identified three adhesion regimes. The film in the complete contact adhesion conforms to the profile of the surface. The adhesive interface in the partial contact adhesion is subdivided into microscopic zones of contact. For glassy adhesion, during peeling, the crack front becomes arrested at the metastable states.^24^

#### 3.2.3 Interfacial Linker

In general, the conventional methods are incompatible to connect two surfaces with special chemical and physical properties.^29^ According to the peeling model, the adhesion energy could be enhanced by simply introducing interlocking linkers, resulting in enhanced adhesive force.^27^, ^28^, ^29^ Eastwood et al.^27^ reported an enhanced attachment between polystyrene/poly(methyl methacrylate) homo­polymers interlocked by a sequence of styrene and methyl methacrylate block copolymers which served as interfacial crosslinkers (Figure 11f). Nanoparticles are another approach to enhance interfacial adhesion. Su et al.^28^ reported that gold nanoparticles modified with low‐molecular‐weight polystyrene ligands could improve the interfacial adhesion of two polystyrene films (Figure 11g). Often, it is difficult to join two surfaces with very low surface energy. Jin et al.^29^ thought of a general solution to make two non‐adhesive surfaces stick together by applying concave tetrapodal linkers (Figure 11h). They also found that the peel strength depended on the shape of the fillers. Compared with convex fillers, tetrapodal shaped particles enhance the peel strength more significantly.^29^

## 4 Conclusion

We summarize the recent advance of the peeling model on how to understand surface adhesion. As a typical model, the peeling model can effectively reflect the dynamical and angle‐dependent adhesive property during attachment and detachment processes, which are limited in other models. With the peeling model, surface adhesion can be roughly controlled by modulating the backing and substrate topography, and/or adding the interfacial linkers. However, there are still some problems to be solved, when using the current peeling model for understanding the surface adhesion effectively. For instance, the peeling model suffers from some limitations for very weak and viscoelastic materials such as soft polymers and tissues, because it neglects extensibility of these materials during peeling process. Surely, the deformation energy has been tentatively considered in the energy equilibrium when large surface extensibility occurs during peeling. Moreover, the peeling model faces challenges in flooded conditions because of the ambiguous contact state between two surfaces. Thus, surface wettability should be considered in peeling model to fit the complex conditions.
